# Proactive tobacco treatment for low income smokers: study protocol of a randomized controlled trial

**DOI:** 10.1186/1471-2458-14-337

**Published:** 2014-04-09

**Authors:** Steven S Fu, Michelle van Ryn, Diana J Burgess, David Nelson, Barbara Clothier, Janet L Thomas, John A Nyman, Anne M Joseph

**Affiliations:** 1 VA HSR&D Center for Chronic Disease Outcomes Research, Minneapolis VA Health Care System, 1 Veterans Drive (152), Minneapolis, MN 55417, USA; 2 Department of Medicine, University of Minnesota Medical School, Minneapolis, MN, USA; 3 Mayo Clinic, Division of Health Care Policy & Research, Rochester, MN, USA; 4 Division of Health Policy and Management, University of Minnesota School of Public Health, Minneapolis, MN, USA

**Keywords:** Smoking cessation, Health care disparities, Low-income population, Minority health

## Abstract

**Background:**

There is a high prevalence of smoking and high burden of tobacco-related diseases among low-income populations. Effective, evidenced-based smoking cessation treatments are available, but low-income smokers are less likely than higher-income smokers to use these treatments, especially the most comprehensive forms that include a combination of pharmacotherapy and intensive behavioral counseling.

**Methods/Design:**

The primary objectives of this randomized controlled trial are to compare the effects of a proactive tobacco treatment intervention compared to usual care on population-level smoking abstinence rates and tobacco treatment utilization rates among a diverse population of low-income smokers, and to determine the cost-effectiveness of proactive tobacco treatment intervention. The proactive care intervention systematically offers low-income smokers free and easy access to evidence-based treatments and has two primary components: (1) proactive outreach to current smokers in the form of mailed invitation materials and telephone calls containing targeted health messages, and (2) facilitated access to free, comprehensive, evidence-based tobacco cessation treatments in the form of NRT and intensive, telephone-based behavioral counseling. The study aims to include a population-based sample (N = 2500) of adult smokers enrolled in the Minnesota Health Care Programs (MHCP), a state-funded health insurance plan for low-income persons. Baseline data is obtained from MHCP administrative databases and a participant survey that is conducted prior to randomization. Outcome data is collected from a follow-up survey conducted 12 months after randomization and MHCP administrative data. The primary outcome is six-month prolonged smoking abstinence at one year and is assessed at the population level. All randomized individuals are asked to complete the follow-up survey, regardless of whether they participated in tobacco treatment. Data analysis of the primary aims will follow intent-to-treat methodology.

**Discussion:**

There is a critical need to increase access to effective tobacco dependence treatments. This randomized trial evaluates the effects of proactive outreach coupled with free NRT and telephone counseling on the population impact of tobacco dependence treatment. If proven to be effective and cost-effective, national dissemination of proactive treatment approaches would reduce tobacco-related morbidity, mortality, and health care costs for low income Americans.

**Clinical trials registration:**

ClinicalTrials.gov: NCT01123967

## Background

Tobacco use rates are alarmingly high in low-income populations. In the United States, 27.9% of adults living below the federal poverty level smoke cigarettes compared to 17% of adults at or above the poverty level [1]. About 34% of adult Medicaid enrollees currently smoke cigarettes, and racial/ethnic minorities and women are disproportionally represented in the Medicaid population [2]. Smoking rates in the uninsured population are similar to the Medicaid population and higher than the general population (32% compared to 16% for ages 18–65) [2]. Smokers with lower incomes are also less likely to use evidence-based smoking cessation treatments such as pharmacotherapy and counseling than smokers with higher incomes [3-8]. In an analysis of the 2000 National Health Interview Survey, among smokers who were attempting to quit, only 15.5% of Medicaid enrollees used a cessation aid compared to 25.4% of individuals with private health insurance [6]. Consequently, low income populations experience an excess burden of tobacco-related morbidity and mortality. Additionally, this group can least afford the cost of cigarettes (an average of $2017 per year in the US) [9].

Over the past decade, coverage for tobacco dependence treatments by federal-state Medicaid programs has improved, but significant limitations remain. For example, the number of state Medicaid programs providing some coverage for tobacco dependence treatment increased from 25 in 1998 [10] to 47 in 2009 [11]. However, in 2009, only 18 state Medicaid programs covered individual cessation counseling for all of their enrollees. Most programs also require co-payments for tobacco dependence treatments (over 70%) and many require prior authorization. In addition, knowledge of Medicaid coverage for tobacco dependence treatments is low among Medicaid enrolled smokers and their physicians. In a 2004 study of enrollees and physicians from two state Medicaid programs with full coverage for nicotine replacement therapy (NRT), only 21% of enrollees and 46% of physicians were aware of this coverage [12]. In a 2003 study of tobacco cessation coverage awareness among New York state Medicaid enrollees, only 7% of those surveyed were aware that their state Medicaid program covered NRT [13].

Both patient-level and provider-level factors contribute to disparities in use of tobacco cessation treatment. Current approaches typically depend on either a smoker’s initiative to actively seek treatment or a clinical encounter in which the provider has the time, willingness, and capacity to deliver quality smoking cessation care [8,14]. Low income smokers may be less likely to seek treatment because of significant life stressors that reduce their motivation to quit, lower levels of knowledge about the benefits of pharmacotherapy as well as unaware that their state Medicaid program covers these treatments [15-17]. In addition, low income smokers experience unique, significant barriers to health care access and are vulnerable to health care providers’ assumptions about lack of interest in quitting [18,19].

Effective strategies are needed to provide increased access to evidence-based tobacco treatments, especially for low-income populations. In this paper, we describe the study design and methods of a prospective randomized controlled trial testing a proactive tobacco intervention that is hypothesized to have greater population impact because it will 1) achieve wide reach and increase utilization of treatment (proactive outreach with offer of free NRT and easy access to telephone care) and 2) increase the effectiveness of treatment (by efficient delivery of free NRT and intensive behavioral counseling). This proactive tobacco treatment intervention integrates population-based and individual approaches to address both patient and provider barriers to providing comprehensive care [20].

## Methods

### Study design

We will examine the population impact of a proactive care intervention in a population-based sample of adult smokers enrolled in the Minnesota Health Care Programs (MHCP). MHCP is a state-funded health insurance plan, administered by the Minnesota Department of Human Services (DHS), for low income persons and families comprising two major publicly subsidized health care assistance programs: Medicaid and MinnesotaCare. MinnesotaCare is for Minnesota residents that do not have access to affordable health care coverage and funded by a state tax on Minnesota hospitals and health care providers, Medicaid and enrollee premiums. The proactive tobacco intervention being tested has two primary components: 1) outreach to current smokers in the form of tailored mailings and telephone calls, and 2) facilitated access to a free, comprehensive, evidence-based treatment for tobacco dependence in the form of NRT and intensive, telephone-based behavioral counseling. This proactive approach is designed to overcome the predominant barriers to smoking cessation treatment (provider-based, access to care, and psychosocial) experienced by low-income smokers. Further, to assess the population impact of smoking cessation treatment, we include all smokers, regardless of their interest in quitting or participation in the offered treatment. This is in contrast to studies designed to test the efficacy of an intervention in smokers who have indicated strong interest in quitting.

**Figure 1:**
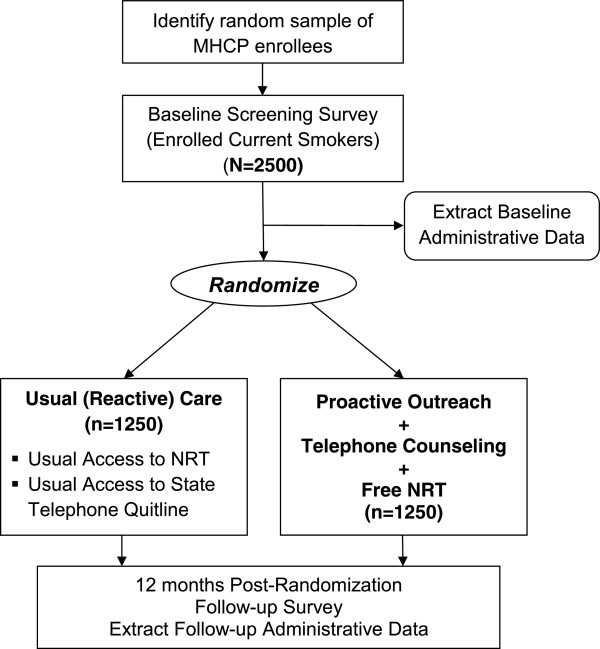
Study design.

The study is a two group randomized controlled trial (see Figure 1) and was approved by the Institutional Review Boards at the University of Minnesota and the Minnesota Department of Human Services. The study population is stratified by age group (18–24, 25–34, and 35–64), by gender, and by healthcare program (Medicaid and MinnesotaCare). Stratified program enrollee data is obtained from MHCP administrative databases and a baseline participant screening survey conducted prior to randomization to assess current tobacco use as well as additional baseline information. Survey recruitment from the strata follows the proportions present in the MHCP enrollee population. Individuals reporting current tobacco use (defined as having smoked a cigarette in the past 30 days, even a puff) on the baseline survey with a valid home address, and an adequate proficiency in English to complete study surveys and participate in telephone counseling are randomized with equal likelihood to receive either 1) proactive outreach combined with free nicotine replacement therapy (NRT) and telephone counseling, or 2) usual (reactive) care available from primary care providers and/or existing telephone helpline programs. The target recruitment goal is 2500 current cigarette smokers.

Outcome data is being collected from a follow-up survey conducted 12 months after randomization as well as from the MHCP administrative data (e.g., pharmacy claims data) for the twelve months following randomization. Since this is a cessation-induction trial (i.e., evaluation of an intervention to encourage cessation among a population-based sample of smokers, including those not currently trying to quit), it is problematic to tie follow-up of cessation outcomes to multiple quit dates. The Society for Research on Nicotine and Tobacco (SRNT) Measures Workgroup recommends that, for cessation-induction trials, follow-ups should be tied to the onset of the intervention, as opposed to aid-to-cessation trials (e.g., efficacy trials of pharmacotherapy) which tie follow-up to the quit date [21].

### Usual care comparison group

All MHCP enrollees are assigned a primary care provider and usual care participants are able to see their provider to access smoking cessation treatment. However, depending on the primary care provider’s willingness and capacity to adhere to guidelines, tobacco treatment is variable, ranging from brief advice and medications to intensive counseling. Usual care participants also have access to smoking cessation medications at substantially reduced cost because MHCP provides insurance coverage for all recommended first-line nicotine replacement products (patch, gum, lozenge, inhaler, and nasal spray), as well as sustained-release bupropion and varenicline. However, an enrollee must obtain a prescription from a provider. Enrollees who fill a prescription may have a co-payment of between $1-$5. Participants can also buy nicotine replacement products out-of-pocket, which are available over-the-counter (i.e., patch, gum, lozenge). Additionally, all residents of Minnesota can access free tobacco treatment counseling from the state quitline (1-888-354-PLAN).

In sum, participants assigned to usual care are able to receive the same or similar smoking cessation treatment components (including pharmacotherapy and telephone counseling) as those given to participants in the proactive care intervention group. What differs is that participants in the proactive intervention are specifically invited – through the methods of proactive outreach -- to consider treatment for tobacco dependence, and given facilitated access to treatment options, whereas participants in the usual care intervention must self-initiate access to treatment.

### Proactive care intervention

The proactive tobacco treatment intervention is based on social cognitive theory (SCT) [22], the stages of change model [23] and the biopsychosocial model of perceived discrimination [24]. Proactive care through mailed invitation materials, outreach calls, and the offer of free NRT along with telephone counseling is expected to largely address provider and patient barriers to initiating care. The proactive care intervention combines two primary components: 1) proactive outreach (a mailed invitation letter followed by telephone outreach) and 2) facilitated access to comprehensive, evidence-based smoking cessation treatment (free telephone counseling and free NRT). Participants are able to choose from one of three over-the-counter NRT products (nicotine patch, gum, or lozenge).

#### *Proactive outreach*

All participants assigned to the proactive care intervention arm are mailed personalized invitation materials. The invitation packet includes a letter and brochure describing the types of tobacco treatment services available to help MHCP enrollees quit smoking. To help them access the available treatment, the materials include instructions for enrollees interested in discussing their smoking to (a) call a toll free number to speak directly with a smoking cessation counselor or (b) return, in a self-addressed stamped envelope, a reply card with contact information.

Three weeks after the mailed invitation materials are sent (or earlier for participants who respond), participants receive an outreach call from a counselor trained in motivational interviewing and smoking cessation treatment. Up to 6 contact attempts are made at different times of the day (i.e., morning, afternoon, evening) during the week. The purpose of the outreach call is to 1) deliver motivational advice to quit smoking, 2) promote self-efficacy, 3) encourage participants to participate in smoking cessation treatment, and 4) provide information on the safety, efficacy, and functional benefits of pharmacotherapy, particularly NRT. However, both a participant’s willingness to engage in this type of discussion and his or her receptivity to treatment-relevant information is variable. Counselors employ motivational interviewing techniques with consideration for the participants’ current stage of change [25]. For example, the counselor asks, “Can we talk about your smoking behavior to see what services might be best for you now or in the future?” Then the counselor assesses the participant’s readiness to quit smoking using a scale from 0 (“no thought of quitting”) to 10 (“taking action to quit”) [26]. The content for the call is then tailored to the smoker’s readiness to quit, individual concerns about quitting, and the associated SCT factors that must be targeted to promote progression through the stages of change, such as self-efficacy and outcome expectations. Because motivational interviewing uses a patient-centered, autonomy-emphasizing approach, rapport may be more easily established, especially among those not highly motivated to quit. Our findings from focus groups with low-income and minority smokers who had negative experiences with being “told” to quit or “scared” into quitting support this approach [27].

#### *Telephone care*

Telephone care in this study combines free proactive phone-based counseling with free NRT. The telephone counseling protocol is similar to that used by state quitlines. Specifically, we use an adaptation of the evidence-based California Helpline protocol, which uses a combination of motivational interviewing and cognitive-behavioral treatment for substance abuse [28]. This standard telephone counseling protocol consists of 7 calls initiated by the counselor, scheduled in a relapse-sensitive fashion over a 2-month period (pre-quit, quit day, then 3 days, 1 week, 2 weeks, 1 months, and 2 months after the quit date). In the pre-quit session, a major portion of the counseling is spent promoting smokers’ self-efficacy using motivational interviewing. Smokers are asked to identify situations in which it would be most difficult to refrain from smoking and to plan realistic coping strategies. During the follow-up calls, the emphasis is on successful implementation of effective coping strategies and relapse prevention. Given variability in participants’ levels of motivation and success with quitting, counseling calls are individually tailored to the participant’s level of motivation and progress. For example, participants who are thinking about quitting but not ready to set a quit date right away, receive motivational interviewing to enhance their readiness to quit. In addition, participants who relapse to smoking are encouraged to set new quit dates using motivational interviewing and repeat the counseling program. In total, a participant is able to receive up to 14 counseling calls.

#### *Free NRT*

The study also provides a free 8-week course of NRT (patch, gum or lozenge), which is mailed directly to participants in anticipation of their quit date. The protocol is based on the US Public Health Service Guideline recommendations [29,30]. All participants who receive telephone counseling are offered NRT unless they have one of the following contraindications: 1) recent (within 2 weeks) heart attack or severe arrhythmia, 2) unstable angina, 3) pregnancy; or they decline medication. If participants are interested in other smoking cessation medications (e.g., bupropion, varenicline), they are referred to their primary care provider to obtain a prescription. Participants who relapse and attempt to quit again are able to receive an additional 4 weeks of NRT.

### Data collection

Data collection occurs at baseline and at 12 months post-randomization. There are three primary sources of individual level data for this study: 1) MHCP administrative data for both pre- and post-randomization periods, 2) participant questionnaire data from baseline and follow-up surveys, and 3) the intervention database (intervention tracking and process data).

#### *Baseline and follow-up survey procedures*

Surveys, particularly of low-income populations, can be susceptible to a low response rate [31,32]. Non-response reduces the effective sample size and can introduce bias and impair the validity and generalizability of results. Mailed surveys assessing Medicaid program members’ quality of care experiences have yielded response rates ranging from 20-50% [33]. A major advantage, however, of using a mailed survey for MHCP enrollees is that there is a strong incentive for enrollees to keep their addresses current in enrollment databases because many receive supplemental income and monthly checks from the government [31].

For the baseline tobacco use survey, we use established modified-Dillman mailed survey procedures [34]. Up to four mailings are sent to the selected participants over the course of five weeks. The first mailing is a pre-notification letter and a reply postcard to return if they have not smoked a cigarette in the past 30 days. The second mailing includes a $2.00 cash incentive, cover letter, the baseline questionnaire, informed consent information, privacy rights information and a self-addressed, business reply envelope. Seven days later, a reminder/thank you post card is sent to potential participants (3rd mailing). Two weeks after the postcard mailing, a replacement questionnaire packet is sent to those who have not yet responded (4th mailing).

The 12 month follow-up survey for all randomized participants follows similar procedures as the baseline survey except that it did not include a pre-notification letter and additional procedures are incorporated in order to reduce attrition. First, instead of a $2.00 incentive, a larger $10.00 incentive is used with the first mailing to promote retention. Second, the follow-up survey includes phone administration (mixed-mode protocol). Approximately five weeks after the initial mailing, participants who did not respond to the mail protocol are contacted for a telephone interview. At least 10 phone call attempts are made at different times of the day and week. Third, tracking procedures for the follow-up survey to reduce attrition are also employed. Using a mixed-mode approach that involves telephone interviews of participants who do not respond to the mail protocol can increase response rates by an additional 5 to 15% [33]. Another advantage of telephone interviews of mail nonrespondents is improved sample representativeness, which potentially reduces nonresponse bias [35].

### Outcomes

The primary outcome is self-reported six-month prolonged abstinence [21] at one year assessed at the population-level (i.e., smoking abstinence among all smokers, including those who use and those who do not use treatment). A participant who smoked at least once on 7 consecutive days or at least once on 2 consecutive weekends in the six-month period is considered a treatment failure. The primary outcome for both groups is measured from the 12 month follow-up survey. Secondary outcomes include self-reported 7-day point prevalence and 30-day point prevalence, defined as having not smoked a part of a cigarette in the past 7 days and as having not smoked a part of a cigarette in the past 30 days, respectively. Following the Society for Research on Nicotine & Tobacco recommendations, biochemical verification of smoking abstinence has not been performed because of the low misreporting rates in large-scale, population based trials that have limited face-to-face contact with study staff [36].

Additional secondary outcomes include utilization of tobacco cessation treatment during the 12 month follow-up (from any source, including care from outside the study) specifically: 1) initiation of combined intensive behavioral counseling and medication treatment, 2) initiation of intensive behavioral counseling, and 3) initiation of medication treatment. These secondary outcomes are primarily assessed using self-report from the follow-up survey because smokers may seek care from outside the study due to the proactive strategies tested. Initiation of medication treatment is defined as using one or more tobacco dependence medications (e.g., NRT, bupropion or varenicline) in the 12 month follow-up period. Initiation of intensive counseling is defined as the completion of at least one call from a telephone counseling program or making at least one visit to a group or individual smoking cessation program in the 12 month follow-up period. In addition, we will assess tobacco dependence medication utilization by extracting pharmacy claims data from the MHCP claims administrative data and examine the prescription rates of individual medications, number of medications prescribed, and duration of medication use.

### Survey measures: potential moderators and confounders

The following participant demographics are collected in the survey: age, gender, race, ethnicity, marital status, United States birth, size of household, number of children in household, education, annual household income, financial stress, and employment status.

#### *Smoking behavior*

Standard questions from the California Tobacco Survey [37], the CDC Behavioral Risk Factor Surveillance System (BRFSS) [38] are used to collect information regarding smoking history, such as age of smoking initiation, previous quit attempts and prior use of tobacco treatment. The Heaviness of Smoking Index is used to assess nicotine dependence [39].

#### *Physical and mental health*

We used instruments from the Patient Reported Outcomes Measurement Information System (PROMIS) developed by the NIH Roadmap initiative to assess physical functioning (short form and global health status) [40]. To assess mental health, we used the PROMIS instruments for anxiety and depression (PHQ-2). To assess alcohol use we used items from the Behavioral Risk Factor Surveillance System (BRFSS) [38] that assess quantity, frequency, and binge drinking. We also used the EQ-5D, which provides a general measure of health status [41].

### Potential mediators

#### *Provider factors*

The provider factors include participants’ perceptions of their primary care provider’s behavior related to delivery of smoking cessation care, and perceived provider bias and cultural competence. HEDIS tobacco performance measures [42] assess participants’ receipt of smoking cessation advice, counseling and treatment from their primary care provider. We used the Physician Bias and Interpersonal Cultural Competence Measures Scale [43] which consists of 3 questions asking about the participant’s being treated with respect by the doctor, the doctor’s understanding of the participant’s background and values, and the participant’s feeling like the doctor looks down on the participant’s way of life. We also assess participant satisfaction with smoking cessation care from their provider [44].

#### *Cognitive factors*

The cognitive factors examined in this study include motivation to quit, self-efficacy, and attitudes toward NRT. The Contemplation Ladder is used to assess motivation to quit and asks participants to indicate their readiness to quit on a scale from 1 to 10 (e.g., “Think I should quit but not quite ready”) [26]. Self-efficacy to quit is assessed with a global measure [45]. We assess beliefs towards NRT using the 12-item Attitudes Towards Nicotine Replacement Therapy (ANRT-12) scale [46]. This scale asks participants to rate their level of agreement or disagreement on the perceived advantages and disadvantages of using NRT. Participants also indicate their degree of certainty regarding whether or not they intend to use NRT during their next quit attempt.

#### *Social environment*

Characteristics of the patient’s social network assessed in the survey include: subjective norms related to smoking, smoking habits of friends and family, and home smoking rules [37]. We also assess day-to-day perceived discrimination experienced by participants due to any cause, not only on the basis of race, using 9 questions that ask about the frequency of exposure to day-to-day experiences of discrimination such as being treated with less courtesy, less respect, or being harassed [47]. Smoking stigma is assessed using an adapted form of the Mental Health Consumers’ Experience of Stigma Scale [48]. This scale asks how frequently a participant has personally experienced sigma, like being viewed unfavorably, avoided, or treated unfairly, because of their smoking status.

### Sample size and power analysis

The power analysis assumes independent samples in the two groups within strata and considers a type one error rate of 0.05 for analysis of the primary outcome, six-month prolonged abstinence. The goal sample size for this study is N = 2500 (1250 per group), which accounts for a 40% nonresponse rate to the follow-up survey to ensure that we have observed smoking status outcome data on 1500 respondents (750 per group). The anticipated six-month prolonged smoking abstinence rate in the usual care arm is between 2% to 5%. This sample size provides over 85% power to detect intervention effects if the intervention raises abstinence rates by 4% and provides approximately 80% power or greater to detect differences if the intervention raises quit rates by 3.5%. The difference this study is powered to detect is of the same relative order of magnitude as those in prior smoking cessation clinical trials (e.g. odds ratios of 1.5 to 2.0) [49].

### Statistical analyses

The primary analysis uses intent-to-treat methodology. The study design is a randomized complete block design with the blocks (or strata) comprising combinations of the two health care programs (Medicaid vs MinnesotaCare), gender, and three age groups (18–24, 25–34, and 35–64). In the analysis we will use stratified logistic regression methods to model the log odds of abstinence (i.e., 6-month prolonged abstinence) or treatment utilization as an additive function of intervention and the healthcare program/age group strata. This regression model is then used to estimate and test intervention effects.

With our anticipated sample size, random assignment is expected to create two groups that are balanced with respect to observed and unobserved baseline characteristics. However, survey nonresponse may introduce substantive imbalance between the intervention and usual care groups. Furthermore, nonresponse may be directly related to smoking status in a non-ignorable manner. To investigate potential differential non-response bias, the respondents in the two groups will be compared with respect to baseline measures a priori known to be related to smoking cessation and treatment utilization. Balance of the two groups will be tested using simple Mantel-Haenszel [2] tests for categorical variables and appropriate parametric tests (e.g. Blocked Anova F-tests) or nonparametric tests (e.g. Wilcoxon rank-sum test) for continuous variables. A common practice within the smoking cessation research community is to treat non-respondents at follow-up as continuing smokers. This practice is perceived to be a conservative approach but does not, in fact, live up to its reputation [50]. To address potential response bias issues, we will implement a propensity-based imputation methodology and pattern-mixture methods in a sensitivity analysis of the primary analyses described above.

#### *Cost-effectiveness analysis*

We will also determine the cost-effectiveness of the intervention compared with usual care. The conduct of the cost-effectiveness analysis will be from the adopter’s perspective, such as the state medical assistance programs or state quitline and tobacco control programs. From this viewpoint, costs are measured by the per participant budgetary costs incurred as a result of implementing the interventions. In practice, this cost concept is similar to that used in many existing cost-effectiveness analyses of smoking cessation programs. The costs to be considered are those related to proactive outreach (mailed invitation materials, telephone outreach), telephone counseling, free NRT, data keeping, materials and overhead. This program perspective does not include indirect costs incurred by individuals. The study also takes care to distinguish those data and processing costs related to the scientific evaluation of the intervention and those related to the intervention itself, and only include the latter in the cost-effectiveness analysis.

The analysis uses the difference in six-month prolonged abstinence as the effectiveness measure. Measures of effectiveness and costs are aggregated and incremental cost-effectiveness ratios (ICERs) will be constructed for the two arms. The ICER takes the form: (C_T_ – C_UC_)/(O_T_ – O_UC_) where (C_T_ – C_UC_) is the expected difference in the costs (C) between the treatment and usual care and (O_T_ –O_UC_) is the corresponding expected difference in the outcome of interest (O), namely the numbers of individuals achieving prolonged abstinence at 12 months. Such ratios can be thought of as measuring the price at which an added unit of the outcome can be purchased by replacing UC with the treatment. We will use bootstrap methods to estimate standard errors and confidence intervals for these cost-effectiveness measures. This approach provides a convenient, nonparametric means of incorporating the correlation of costs and outcomes into the resulting standard errors. Sources of uncertainty in the specification of components of the cost measures are addressed by employing probabilistic sensitivity analyses, where the distributions of the variables representing the analytical assumptions are allowed to vary simultaneously, thus testing the robustness of our findings [51]. This analysis is repeated for the different sets of sensitivity analyses described above. In these analyses the total costs of the intervention do not change but the effectiveness measures changes with the different assumptions.

### Discussion

There is clear evidence that low-income smokers are less likely than higher-income smokers to use tobacco dependence treatments, especially the most comprehensive form that includes a combination of pharmacotherapy and intensive behavioral counseling. Therefore, there is a critical need to increase access to effective treatment and lessen the burden of tobacco related disease among low income populations, due to the high prevalence of smoking. Telephone quitline services are the backbone of universal access to tobacco dependence treatment and are critical to the provision of smoking cessation services to the poor and uninsured. Quitlines, however, continue to be under-used and do not consistently provide comprehensive care (medications with counseling). In this randomized controlled trial, we are testing an innovative intervention that integrates population-based and individual approaches to address both patient and provider barriers to treatment that low-income smokers face. This trial will provide evidence regarding the effects of proactive outreach coupled with free NRT and telephone counseling on the population impact of tobacco dependence treatment. Results of this work have the potential to transform recommendations for promoting national and state delivery of tobacco treatment to low-income populations.

## Competing interests

The authors declare that they have no competing interests.

## Authors’ contributions

SSF, AMJ and MVR conceived the study design and drafted the study protocol. DJB, DN, BC, JT, JN helped to draft the study protocol. All authors provided critical review of the study protocol and approved the final manuscript.

## Pre-publication history

The pre-publication history for this paper can be accessed here:

http://www.biomedcentral.com/1471-2458/14/337/prepub
